# Supplementary material for: The Chromosome-level Genome of the Ctenophore Mnemiopsis leidyi A. Agassiz, 1865 Reveals a Unique Immune Gene Repertoire
Source: Genome Biol Evol. 2025 Jan 21;17(2):evaf006. doi: 10.1093/gbe/evaf006 (PMC11797021; doi:10.1093/gbe/evaf006)

Supplementary Material

Supplementary Figure 1. Chromosome-level assembly. Heat maps of Omni-C vs. HiRise alignment for the genome of *Mnemipsis leidyi*.

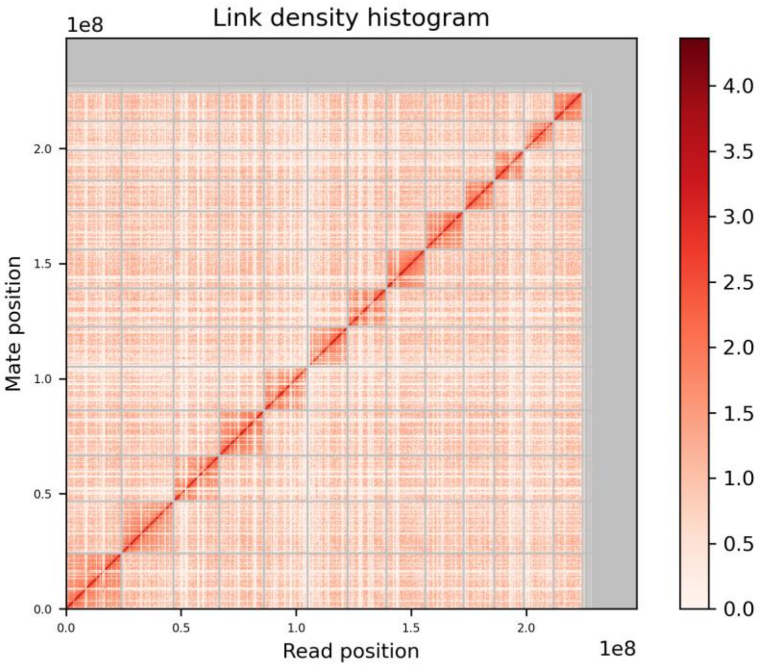

Supplementary Figure 2. Synteny between 3 ctenophore species based on metazoan BUSCO hits. Synteny is conserved among *Mnemipsis leidyi*, *Bolinopsis microptera* and *Hormophora californensis*. *M. leidyi* scaffolds have been relabelled accordingly: c1 [scaffold_6], c2 [scaffold_3], c3 [scaffold_2], c4 [scaffold_5], c5 [scaffold_1], c6 [scaffold_7], c7 [scaffold_11], c8 [scaffold_8], c9 [scaffold_12], c10 [scaffold_9], c11 [scaffold_4], c12 [scaffold_13], c13 [scaffold_10].

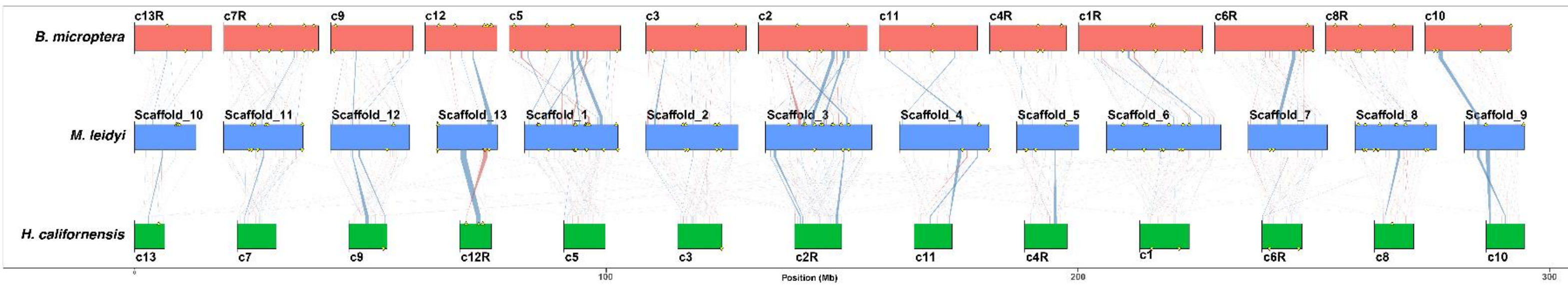

Supplement: evaf006_Supplementary_Data [file evaf006_supplementary_data.zip › Supplementary_Figures.pdf]
